# Enhanced Tear Film Concentrations of Cefazolin and Chloramphenicol Using Cross‐Linked Hyaluronic Acid in Canine Eyes

**DOI:** 10.1111/vop.70013

**Published:** 2025-03-16

**Authors:** Dikla Arad, Ella Margot Mordechai, Yulia Goncharov, Ron Ofri, Lionel Sebbag

**Affiliations:** ^1^ Koret School of Veterinary Medicine The Hebrew University of Jerusalem Rehovot Israel

**Keywords:** antibiotic, bacterial keratitis, eye drop, mucoadhesive polymer, pharmacokinetics, tear film

## Abstract

**Objective:**

To evaluate the impact of two excipients, 1.4% polyvinyl alcohol (PVA) and 0.75% cross‐linked hyaluronic acid (XHA), on tear film concentrations of cefazolin and chloramphenicol.

**Animals Studied:**

Ten ophthalmologically healthy dogs.

**Procedures:**

Cefazolin and chloramphenicol were compounded into 5.5% and 0.5% solutions, respectively, using either 1.4% PVA or 0.75% XHA. In the first trial, each dog received cefazolin‐PVA in one randomly assigned eye and cefazolin‐XHA in the contralateral eye. One month later, the experiment was repeated using chloramphenicol formulations. Tear fluid was sampled at 0, 1, 5, 10, 15, 30, 60, 120, 240, 360, and 480 min following eyedrop administration using 2 μL capillary tubes. Tear concentrations of cefazolin and chloramphenicol were measured using UV–Vis spectrophotometry.

**Results:**

Tear film concentrations of cefazolin and chloramphenicol were significantly higher with XHA compared to PVA at all time points (*p* ≤ 0.020), except for baseline (both antibiotics), times 1 min, 60 min, and 120 min for cefazolin. The tear film kinetics exhibited a biphasic pattern, with drug levels decreasing from 0 to 120 min, then slightly increasing between 120 and 360 min before declining again until 480 min. The area under the time‐concentration curve (AUC_0−480_) was significantly greater with XHA versus PVA formulations (*p* = 0.002), with a median 2.4 and 4.2 times higher for cefazolin and chloramphenicol, respectively.

**Conclusion:**

The cross‐linked hyaluronic acid significantly enhanced the retention and overall exposure of both cefazolin and chloramphenicol in the canine tear film. These findings suggest that XHA could serve as a superior delivery vehicle for ocular antibiotics, potentially improving treatment outcomes for ophthalmic infections.

## 1 Introduction

Ophthalmic solutions (eyedrops) are the most common formulation for managing ocular diseases in both human and veterinary medicine [1]. Eyedrops offer ease of use, convenience, and high drug levels in the target tissue while minimizing systemic side effects [1]. However, their bioavailability is often limited by ocular barriers (e.g., blood‐tear barrier, corneal barrier) and physiological processes like reflex tearing, blinking, and nasolacrimal drainage that rapidly clear the solution to maintain ocular surface homeostasis [1, 2, 3]. Recent studies in dogs have demonstrated a 20%–45% decrease in tear film drug levels within 1 min and an 80%–95% decrease by 15 min post‐administration [4, 5].

Several strategies can be employed to optimize ocular drug delivery with eyedrops, such as repeated eyedrop administration [6], higher drug concentrations [6], and mucoadhesive polymers to prolong contact time on the ocular surface [7, 8, 9]. Hyaluronic acid (HA) is a natural mucoadhesive polymer that is commonly used in ophthalmology as the main ingredient of lubricants, subdermal fillers, and viscoelastics [10]. Notably, HA has demonstrated benefits when combined with antimicrobial drugs. For example, gentamicin combined with HA increased antibiotic concentrations in the tear film of human subjects [9], and amikacin combined with HA achieved higher antibiotic levels in the lacrimal fluid of rabbits [8]. However, these findings cannot be directly extrapolated across species due to differences in ocular surface anatomy and tear film dynamics.

The benefits of HA are further enhanced by cross‐linking technology, whereby changes in physicochemical properties (e.g., high viscosity, non‐Newtonian rheology, resistance to enzymatic degradation) provide cross‐linked hyaluronan (XHA) with prolonged precorneal contact time and improved spread over the ocular surface during blinking [10, 11, 12, 13]. XHA's enhanced viscosity and mucoadhesive properties seem to be a promising candidate for improved ocular drug delivery. Therefore, this study investigated XHA as a potential tool to enhance antibiotic delivery to the ocular surface in canine patients. Cefazolin and chloramphenicol were selected for their broad‐spectrum activity against common ocular pathogens [14] and lack of commercial availability in certain markets, necessitating compounded preparation. Given the established benefits of combining linear HA with antibiotics and the advantages of cross‐linking, we hypothesized that XHA would significantly improve the tear film pharmacokinetics of these antibiotics compared to standard compounding with polyvinyl alcohol‐based artificial tears.

## 2 Material and Methods

### 2.1 Animals

Based on tear film levels of topical antibiotics in canines [15], assuming a 30% difference in tear concentrations with the addition of hyaluronic acid (mean difference 60 μg/mL, standard deviation 58 μg/mL) [9], a power of 80% and a significance level (α) of 0.05, a sample of *n* = 10 dogs was deemed sufficient to obtain significant differences between groups (paired *t* tests; SigmaPlot version 15; Systat Software Inc.). Ten dogs (20 eyes) were included in the study, including five brachycephalic and five nonbrachycephalic dogs. Prior to study inclusion, each dog underwent a complete physical examination and ophthalmic examination by a board‐certified veterinary ophthalmologist (L.S.), including Schirmer tear test‐1 (STT‐1; Ophthalmic strips, IMS Euro Ltd), fluorescein staining of the ocular surface (AKti‐flu strips, AKtive Srl), slit‐lamp biomicroscopy (SL‐17, Kowa) and indirect ophthalmoscopy (Heine Omega 500). Exclusion criteria included STT‐1 values < 15 mm/min, ocular pathology (excluding minor incidental findings), or systemic illness. The study was approved by the Hebrew University of Jerusalem's Institutional Animal Care and Use Committee (protocol # MD‐23‐17 182‐2), and the experiments adhered to the Guidelines for Ethical Research in Veterinary Ophthalmology (GERVO).

### 2.2 Antibiotics Compounding

Cefazolin was prepared as a 5.5% ophthalmic solution by mixing cefazolin 1 g (Cefazolin sodium, Trima Ltd) with 10 mL of sterile water for injection (Hospira Inc., Lake Forest), then mixing 2.75 mL of the 10% cefazolin solution with either 2.25 mL of 1.4% polyvinyl alcohol (PVA; Refresh, Allergan) or 2.25 mL of 0.75% cross‐linked hyaluronan (XHA; Oculenis, Sentrx Animal Care) to provide 5.5% cefazolin‐PVA and 5.5% cefazolin‐XHA solutions, respectively.

Chloramphenicol was prepared as a 0.5% ophthalmic solution by mixing chloramphenicol 1 g (Chloramphenicol sodium succinate, VUAB Pharma) with 10 mL of sterile water for injection, then mixing 0.25 mL of the 10% cefazolin solution with either 4.75 mL of 1.4% polyvinyl alcohol (PVA; Refresh, Allergan) or 4.75 mL of 0.75% cross‐linked hyaluronan (XHA; Oculenis, Sentrx Animal Care) to provide 0.5% chloramphenicol‐PVA and 0.5% chloramphenicol‐XHA solutions, respectively. All formulations were compounded aseptically by a pharmacist into standardized 10 mL eyedrop bottles (Weener Empire Plastics Pvt. Ltd), prepared fresh the day before each experimental day.

In the first trial, dogs were treated with one drop of cefazolin‐PVA in one randomly selected eye and one drop of cefazolin‐XHA in the contralateral eye. One month later, the same design was repeated with chloramphenicol.

### 2.3 Tear Collection and Drug Quantification

Tear samples were collected with 2 μL capillary glass tubes at *t* = 0 min (i.e., immediately after eyedrops administration and spontaneous blinking), then 1, 5, 10, 15, 30, 60, 120, 240, 360, and 480 min later. Following the establishment of standard curves using known concentrations (0.01–30 000 μg/mL) of cefazolin standard (Cefazolin sodium USP reference standard, Sigma Aldrich; *R* ^2^ = 0.998) and chloramphenicol standard (Chloramphenicol USP reference standard, Sigma Aldrich; *R* ^2^ = 0.999), antibiotic concentrations were measured in all tear samples using UV–Vis spectrophotometry (NanoDrop One, Thermo Fischer Scientific Inc.) at 270‐nm absorbance for cefazolin and 278‐nm absorbance for chloramphenicol, as previously described [16, 17].

### 2.4 Physicochemical Properties

For each antibiotic, the following outcomes were compared between XHA alone (control) and antibiotic–XHA, compounded as described above.

#### 2.4.1 Drop Size

Using a similar 10‐mL bottle, a single drop of each formulation (control, cefazolin‐XHA, chloramphenicol‐XHA) was individually dispensed into a sterile plate, then the drop volume was measured with a pipette (Eppendorf Reference 2, 10–100 μL). This experiment was repeated 60 times (i.e., 60 individual drops) to calculate the average and standard deviation of the drop size (in μL) for each formulation.

#### 2.4.2 pH

The pH of each formulation was measured using a benchtop pH meter (Orion Star A211, Thermo Fischer Scientific Inc.).

#### 2.4.3 Viscosity

The viscosity profile was measured in Pa*s with a rheometer (Discovery HR 20, TA Instruments) at a shear rate of 2.5 s^−1^ as well as a range from 0 to 20 s^−1^.

#### 2.4.4 Stability

The absorbance of each cefazolin and chloramphenicol formulation was measured daily over a 30‐day time period in both XHA or phosphate‐buffered saline (control). Samples were held at 4°C without humidity control.

### 2.5 Data Analysis

Normality of the data was evaluated with the Shapiro–Wilk test; data was not normally distributed for cefazolin (XHA, *p* < 0.001; PVA, *p* < 0.001) or chloramphenicol (XHA, *p* < 0.001; PVA, *p* < 0.001). For each antibiotic, Mann Whitney tests were used to compare drop sizes and tear film concentrations between brachycephalic vs. nonbrachycephalic dogs in eyes receiving PVA and in eyes receiving XHA; since no statistical differences were observed for either cefazolin (*p* ≥ 0.095) or chloramphenicol (*p* ≥ 0.095), data from all *n* = 10 dogs were used for further analysis. For each antibiotic, a linear mixed‐effects model was conducted, incorporating ‘dog’ as a random effect and ‘time’ and ‘treatment’ (XHA or PVA) as fixed effects. Further, Wilcoxon signed‐rank tests were used to compare PVA‐eyes and XHA‐eyes for tear film concentrations at each time point (0 min to 480 min) and for the area under the concentration‐time curve (AUC _0–480_), calculated using the linear trapezoidal rule. Statistical analysis was performed using R software version 4.4.2 (mixed‐effects model) and SigmaPlot version 15.0 (Systat Software Inc.), with *p* values lower than 0.05 considered significant.

## 3 Results

The study population comprised five brachycephalic dogs (2 Shih Tzus, 1 French Bulldog, 1 Pekingese, 1 Boxer) and five nonbrachycephalic dogs (4 mixed breeds, 1 Malinois), including 6 castrated males and 4 spayed females. Mean ± standard deviation (min‐max) age and body weight were 4.1 ± 3.8 [1, 2, 3, 4, 5, 6, 7, 8, 9, 10, 11, 12, 13, 14] years and 19.1 ± 9.1 (6.6–35) kg, respectively. Descriptive data for tear film concentrations of each antibiotic are reported in Appendix S1.

### 3.1 Cefazolin

**FIGURE 1:**
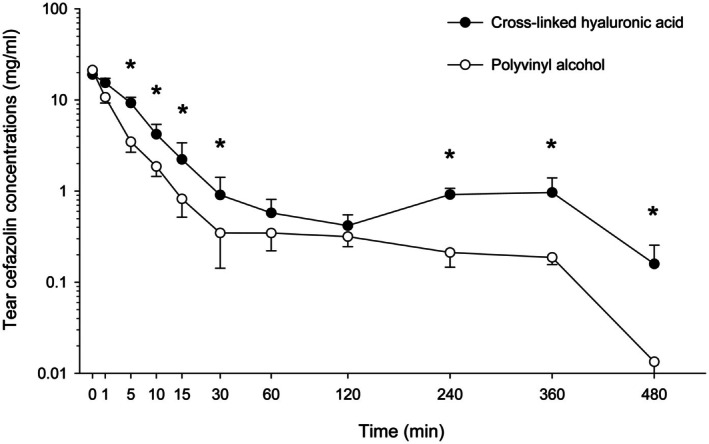
Scatter plot depicting the median ± SEM of tear cefazolin concentrations over time in ten dogs receiving 5.5% cefazolin compounded in polyvinyl alcohol (one eye; white circles) or cross‐linked hyaluronic acid (other eye; black circles). The *y* axis is displayed in logarithmic scale. Asterisks (*) indicate statistical differences (*p* < 0.05) between eyes at each time point.

Tear film concentrations of cefazolin are summarized in Figure 1. As shown in Figure 1, tear film concentrations of cefazolin were significantly higher in XHA versus PVA eyes at all time points (*p* ≤ 0.008) except for baseline (*p* = 0.695), *t* = 1 min (*p* = 0.084), *t* = 60 min (*p* = 0.322) and *t* = 120 min (*p* = 0.275). Further, median AUC_0–480_ was 2.4‐fold higher in eyes receiving XHA (514.3 mg/mL × min) compared to PVA (210.7 mg/mL × min), a finding that was statistically significant (*p* = 0.002). The linear mixed‐effects model confirmed that XHA was superior to PVA, with significant effects of time (Estimate: −5.236; *p* < 0.001) and the interaction between treatment and time (Estimate: 1.727; *p* = 0.041) on cefazolin concentrations. Variability due to individual dogs was negligible (Std. Dev. = 0.0), indicating consistent trends across subjects.

### 3.2 Chloramphenicol

**FIGURE 2:**
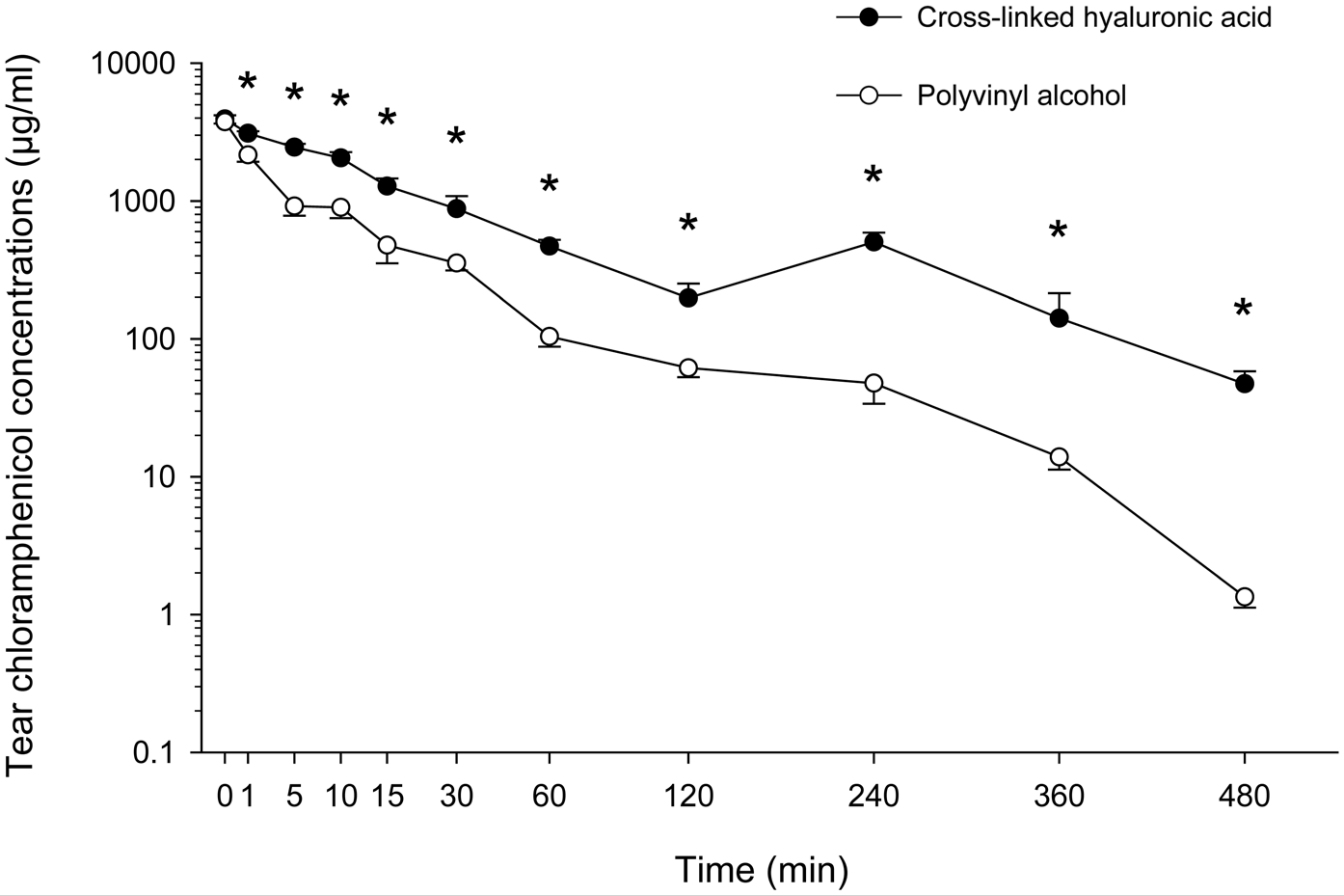
Scatter plot depicting the median ± SEM of tear cefazolin concentrations over time in ten dogs receiving 0.5% chloramphenicol compounded in polyvinyl alcohol (one eye; white circles) or cross‐linked hyaluronic acid (other eye; black circles). The *y* axis is displayed in logarithmic scale. Asterisks (*) indicate statistical differences (*p* < 0.05) between eyes at each time point.

Tear film concentrations of chloramphenicol are summarized in Figure 2. As shown in Figure 2, tear film concentrations of chloramphenicol were significantly higher in XHA versus PVA eyes at all‐time points (*p* ≤ 0.020) except for baseline (*p* = 0.577). Further, median AUC_0–480_ was 4.2‐fold higher in eyes receiving XHA (205.6 mg/mL × min) compared to PVA (49.0 mg/mL × min), a finding that was statistically significant (*p =* 0.002). The linear mixed‐effects model demonstrated that XHA was superior to PVA, with significant effects of treatment (Estimate: −803.297; *p* < 0.001), time (Estimate: −5.236; *p* < 0.001), and the interaction between treatment and time (Estimate: 1.727; *p* = 0.041) on chloramphenicol concentrations. Variability due to individual dogs was negligible (Std. Dev. = 0.0).

Tear clearance was somewhat ‘biphasic’ in XHA eyes (but not PVA eyes), with tear antibiotic levels initially decreasing from 0 to 120 min, then slightly increasing from 120 to 240 min (chloramphenicol) or 360 min (cefazolin), then decreasing again until the last time point (480 min).

### 3.3 Physicochemical Properties

#### 3.3.1 Drop Size

Median (± SEM) drop size was significantly higher for cefazolin‐XHA versus cefazolin‐PVA (45.9 ± 0.4 μL vs. 40.0 ± 0.3 μL respectively; *p* < 0.001) and for chloramphenicol‐XHA versus chloramphenicol‐PVA (45.7 ± 0.4 μL vs. 40.1 ± 0.3 μL respectively; *p* < 0.001).

#### 3.3.2 pH

The pH was 7.15 for XHA alone, 7.13 for chloramphenicol‐XHA, and 6.54 for cefazolin‐XHA.

#### 3.3.3 Viscosity

The viscosity exhibited similar profiles over 0.1–20 s^−1^ for all formulations evaluated. At 2.5 s^−1^, the shear rate was calculated as 2.027 Pa*s for XHA alone, 2.039 Pa*s for chloramphenicol‐XHA, and 1.87 Pa*s for XHA‐cefazolin.

#### 3.3.4 Stability

The peak absorbance for chloramphenicol and cefazolin was 276 nm and 272 nm, respectively. Following the compounding of each antibiotic with XHA, the drug concentration remained within 90%–110% of the initial concentration (i.e., 0.5% and 5.5% for chloramphenicol‐XHA and cefazolin‐XHA, respectively) over a 30‐day period when the eyedrop bottles were stored under refrigerated conditions.

## 4 Discussion

The present study provides compelling evidence for the superior ocular delivery of topical antibiotics when formulated with XHA versus PVA‐based lubricant. This enhancement in drug delivery was observed consistently in both brachycephalic and nonbrachycephalic dogs; the absence of differences in tear film pharmacokinetics between the two cephalic conformations corroborates previous findings on ciprofloxacin [15] and likely reflects the similar tear film dynamics across canine breeds, such as tear volume and turnover rate [3]. With XHA, tear film concentrations of chloramphenicol were significantly greater at all assessed time points, while cefazolin concentrations were elevated at most time points following a single eyedrop administration. These results are consistent with prior research demonstrating that HA enhances the ocular bioavailability of antibiotics [8, 9]. Interestingly, the greater pharmacokinetic benefits observed for chloramphenicol compared to cefazolin, particularly the higher AUC relative to control, may be attributed to the higher concentration of XHA in the compounded formulation since the antibiotic concentration was lower with chloramphenicol (0.5%) than cefazolin (5.5%).

The average drop size for the XHA formulation was approximately 14% larger than that for PVA when dispensed with the same eyedropper, a difference likely attributable to XHA's increased viscosity and surface tension properties [10]. However, this variation in drop size does not fully account for the observed differences in antibiotic concentrations, as baseline concentrations immediately following instillation were not significantly different between the two formulations. For cefazolin (but not chloramphenicol), the addition of XHA slightly reduced the pH (6.54 vs. 7.23) and viscosity (1.86 Pa*s vs. 2.48 Pa*s) compared to the PVA formulation, although these values remained well within the product specifications (pH: 6–7.5; viscosity: 1–6 Pa*s). Importantly, the antibiotics demonstrated stability over 30 days under refrigerated conditions when compounded with XHA, with concentrations remaining between 90%–110% of baseline levels across all evaluated time points.

This study is unique in its application of XHA, a chemically modified formulation that offers enhanced physicochemical properties compared to linear HA [10, 11, 12, 13]. The mechanism by which XHA augments tear film concentrations of antibiotics is likely multifaceted. XHA's increased viscosity and non‐Newtonian rheological behavior could prolong precorneal contact time, thereby enhancing drug penetration across the ocular surface. Additionally, the mucoadhesive properties of XHA may facilitate drug binding to ocular mucins, further enhancing retention time. The observed biphasic clearance pattern in XHA‐treated eyes, with a secondary increase in drug levels following the initial decline, suggests a potential reservoir effect. This effect may be due to the accumulation of XHA in the medial canthus [13], allowing for subsequent redistribution of the antibiotic following blinking. Collectively, these findings suggest that the enhanced tear film concentrations and prolonged drug exposure achieved with XHA formulations may allow for a reduced dosing frequency of topical antibiotics, thereby potentially improving owner compliance and clinical outcomes in the management of bacterial keratitis. In addition to its pharmacokinetic advantages, XHA may offer further clinical benefits. High molecular‐weight forms of HA, such as XHA, exhibit inherent antibacterial properties that inhibit bacterial adhesion and impede biofilm formation [18, 19, 20], which are critical factors in the pathogenesis of bacterial infections. Moreover, HA has demonstrated antimicrobial activity against a broad spectrum of pathogens, including fungi and viruses [20, 21], suggesting its potential as a versatile adjunct in ocular therapy. Last, XHA has been shown to accelerate corneal wound healing [22, 23, 24, 25] and improve tear film quality [26], thereby facilitating the rapid restoration of ocular surface homeostasis.

While our study demonstrates the pharmacokinetic advantages of XHA as an excipient for topical antibiotics, further research is warranted to evaluate its clinical efficacy and optimal dosing strategies for treating bacterial keratitis in canine patients. Future investigations should determine the minimal inhibitory concentrations (MICs) of XHA‐antibiotic formulations against common canine bacterial pathogens (e.g., *Staphylococcus pseudintermedius* , *Streptococcus canis* , *Pseudomonas aeruginosa* ) [14], using growth media supplemented with serum albumin to enhance clinical relevance [27]. These MIC data could then be used to calculate pharmacokinetic‐pharmacodynamic (PK‐PD) indices such as the percent time above MIC (%T>MIC) and ratio of area under the curve to MIC (AUC:MIC) [28, 29]. For time‐dependent antibiotics like cefazolin, %T>MIC is the most relevant PK‐PD target as cefazolin demonstrates maximal efficacy when drug concentrations exceed the MIC [29]. In contrast, the PK‐PD indices for chloramphenicol are less well defined given that its antimicrobial activity can vary between bacteriostatic and bactericidal effects depending on the bacterial species and drug concentrations at the infection site [30]; consequently, both %T>MIC and AUC:MIC may be relevant targets for optimizing chloramphenicol therapy [31]. On a separate note, since XHA is unavailable in some countries, further research on linear HA and other mucoadhesive polymers is needed. Madruga et al. showed that 0.15% linear HA improved tear retention compared to 0.5% carboxymethylcellulose in dogs with keratoconjunctivitis sicca but not in healthy dogs [32]. Similarly, Arad et al. reported no significant differences between 1.4% hydroxyethyl cellulose and 1.2% linear HA in improving tropicamide absorption [7]. These findings highlight the need for accessible and cost‐effective alternatives for ocular drug delivery in settings where XHA is not available.

Several limitations of this study should be taken into account when interpreting the findings. First, the relatively small sample size may restrict the generalizability of the results to larger or more diverse populations; despite efforts to include a diverse sample encompassing both brachycephalic and non‐brachycephalic dogs, the findings may not fully represent specific subgroups such as individual canine breeds. Second, the present study was limited to healthy canine subjects without ocular pathology; future studies should include dogs with altered tear film dynamics or disrupted blood‐tear barriers to assess the performance of XHA‐antibiotic formulations in a more clinically relevant context [33, 34]. The study also relied on compounded formulations of ophthalmic antibiotics, which can present challenges in terms of stability and consistency [35]. When a given antibiotic is commercially available, separate administration of XHA may serve as a viable alternative to compounding, as demonstrated in a recent proof‐of‐concept study by Arad et al. [7].

In conclusion, this study demonstrates that cross‐linked hyaluronic acid significantly enhances tear film concentrations of cefazolin and chloramphenicol, indicating its potential as an effective vehicle for ocular antibiotic delivery. Further exploration is warranted to evaluate the application of XHA as a delivery vehicle for other ophthalmic drugs, such as antivirals, antifungals, and anti‐collagenolytics.

## Author Contributions

**Dikla Arad:** writing – original draft, writing – review and editing, conceptualization. **Ella Margot Mordechai:** data curation. **Yulia Goncharov:** data curation. **Ron Ofri:** methodology, writing – review and editing. **Lionel Sebbag:** methodology, supervision, writing – review and editing, formal analysis, funding acquisition, data curation, conceptualization.

## Ethics Statement

This study was approved by the Hebrew University of Jerusalem's Institutional Animal Care and Use Committee (MD‐23‐17 182‐2).

## Conflicts of Interest

The corresponding author (L.S.) serves on the scientific advisory committee for Sentrx Animal Care, a Dômes Pharma company. The funders of the study had no role in study design, data collection, data analysis, data interpretation, or writing of the report. The corresponding author had full access to all the data in the study and had final responsibility for the decision to submit for publication.

## Supporting information


Appendix S1.

## Data Availability

The data that support the findings of this study are available on request from the corresponding author. The data are not publicly available due to privacy or ethical restrictions.
