# Supplementary material for: Enhanced Tear Film Concentrations of Cefazolin and Chloramphenicol Using Cross‐Linked Hyaluronic Acid in Canine Eyes
Source: Vet Ophthalmol. 2025 Mar 16;29(1):e70013. doi: 10.1111/vop.70013 (PMC12761352; doi:10.1111/vop.70013)
Supplement: Supplementary file 1 — Appendix S1. [file VOP-29-0-s001.docx]

| Time  (min) | Cefazolin-XHA  (µg/ml) | Cefazolin-PVA  (µg/ml) | *P* value  (Wilcoxon test) |
| --- | --- | --- | --- |
| 0 | 19032 ± 1886  (14464 – 30291) | 21254 ± 2560  (6985 – 30387) | 0.695 |
| 1 | 15421 ± 1878  (6203 – 26424) | 10711 ± 1455  (5227 – 19271) | 0.084 |
| 5 | 9249 ± 1436  (2327 – 17490) | 3470 ± 797  (1421 – 7801) | 0.006 |
| 10 | 4206 ± 1196  (959 – 14526) | 1863 ± 410  (668 – 3935) | 0.004 |
| 15 | 2225 ± 1163  (1090 – 12366) | 823 ± 305  (325 – 3495) | 0.008 |
| 30 | 908 ± 508  (378 – 5835) | 347 ± 204  (150 – 2209) | 0.004 |
| 60 | 577 ± 233  (109 – 2490) | 346 ± 125  (236 – 1470) | 0.322 |
| 120 | 418 ± 132  (173 – 1262) | 316 ± 71  (62 – 843) | 0.275 |
| 240 | 919 ± 155  (301 – 2085) | 211 ± 66  (109 – 762) | 0.002 |
| 360 | 966 ± 431  (217 – 5003) | 187 ± 30  (0 – 292) | 0.002 |
| 480 | 159 ± 96  (22 – 1073) | 13 ± 11  (0 – 101) | 0.004 |

**Appendix**

**Appendix Table 1.** Median ± standard error of the mean (minimum – maximum) tear cefazolin concentrations in 10 dogs receiving 5.5% cefazolin compounded in polyvinyl alcohol in one eye (PVA) or cross-linked hyaluronic acid in the other eye (XHA).

**Appendix Table 2.** Median ± standard error of the mean (minimum – maximum) tear chloramphenicol concentrations in 10 dogs receiving 0.5% cefazolin compounded in polyvinyl alcohol in one eye (PVA) or cross-linked hyaluronic acid in the other eye (XHA).

| Time  (min) | Chloramphenicol-XHA  (µg/ml) | Chloramphenicol-PVA  (µg/ml) | *P* value  (Wilcoxon test) |
| --- | --- | --- | --- |
| 0 | 3938 ± 250  (2835 – 5333) | 3749 ± 98  (3107 – 3934) | 0.577 |
| 1 | 3099 ± 113  (2427 – 3628) | 2157 ± 232  (914 – 2958) | 0.010 |
| 5 | 2458 ± 144  (1707 – 3241) | 917 ± 136  (446 – 2043) | 0.004 |
| 10 | 2052 ± 214  (794 – 2916) | 898 ± 148  (236 – 1605) | 0.014 |
| 15 | 1281 ± 174  (756 – 2527) | 477 ± 124  (77 – 1513) | 0.020 |
| 30 | 879 ± 206  (279 – 2162) | 354 ± 41  (184 – 590) | 0.004 |
| 60 | 469 ± 54  (221 – 750) | 104 ± 16  (44 – 213) | 0.002 |
| 120 | 198 ± 53  (69 – 562) | 62 ± 9  (0 – 79) | 0.002 |
| 240 | 503 ± 86  (41 – 869) | 48 ± 14  (10 – 135) | 0.002 |
| 360 | 141 ± 72  (47 – 674) | 14 ± 3  (4 – 29) | 0.002 |
| 480 | 47 ± 11  (13 – 116) | 1 ± 0  (1 – 3) | 0.002 |
